# Supplementary material for: Tuberculosis care provided by private practitioners in an urban setting in Indonesia: Findings from a standardized patient study
Source: PLOS Glob Public Health. 2024 Jun 4;4(6):e0003311. doi: 10.1371/journal.pgph.0003311 (PMC11149835; doi:10.1371/journal.pgph.0003311)
Supplement: S1 Table — (DOCX) [file pgph.0003311.s001.docx]

**S1 Table: Main outcomes of standardized patient visits to private and public practitioners, according to clinical case scenario and the types of providers**.
Scenarios B and C were not presented to general practitioners in the community health centers. Percentages and 95% confidence intervals (CIs) are calculated from the number of encounters.

|  | Community Health Centers | |  | General Practitioners | |  | Specialists | |
| --- | --- | --- | --- | --- | --- | --- | --- | --- |
|  | % | 95% CI |  | % | 95% CI |  | % | 95% CI |
| **Scenario A** |  |  |  |  |  |  |  |  |
| Number of visits (n) | 30 |  |  | 52 |  |  | 15 |  |
| *Adherence to guideline* |  |  |  |  |  |  |  |  |
| NTP guideline | 86.7 | 69.3-96.2 |  | 30.8 | 18.7-45.1 |  | 20.0 | 4.3-48.1 |
| +CXR | 86.7 | 69.3-96.2 |  | 67.3 | 52.9-79.7 |  | 86.7 | 59.5-98.3 |
| *Recommended diagnostic test* |  |  |  |  |  |  |  |  |
| Sputum microscopy | 86.7 | 69.3-96.2 |  | 30.8 | 18.7-45.1 |  | 20.0 | 4.3-48.1 |
| Xpert | 0.0 |  |  | 0.0 |  |  | 0.0 |  |
| CXR | 3.3 | 0.1-17.2 |  | 59.6 | 45.1-72.9 |  | 86.7 | 59.5-98.3 |
| *Prescription* |  |  |  |  |  |  |  |  |
| Anti-TB, correct regimen | 0.0 |  |  | 0.0 |  |  | 6.7 | 0.2-31.9 |
| Anti-TB, incorrect regimen | 0.0 |  |  | 3.9 | 0.5-13.2 |  | 13.3 | 1.7-40.5 |
| Other antibiotics | 30.0 | 14.7-49.4 |  | 76.9 | 63.2-87.5 |  | 53.3 | 26.6-78.7 |
| Anti-TB + other antibiotics | 0.0 |  |  | 0.0 |  |  | 6.7 | 0.2-31.9 |
| Corticosteroids | 10.0 | 2.1-26.5 |  | 30.8 | 18.7-45.1 |  | 13.3 | 1.7-40.5 |
| **Scenario B** |  |  |  |  |  |  |  |  |
| Number of visits (n) |  |  |  | 60 |  |  | 18 |  |
| *Adherence to guideline* |  |  |  |  |  |  |  |  |
| NTP guideline |  |  |  | 88.3 | 77.4-95.2 |  | 88.9 | 65.3-98.6 |
| +CXR |  |  |  | 88.3 | 77.4-95.2 |  | 88.9 | 65.3-98.6 |
| *Recommended diagnostic test* |  |  |  |  | *-* |  |  |  |
| Sputum microscopy |  |  |  | 11.7 | 4.8-22.6 |  | 11.1 | 1.4-34.7 |
| Xpert |  |  |  | 0.0 | - |  | 0.0 |  |
| CXR |  |  |  | 71.7 | 58.6-82.6 |  | 77.8 | 52.4-93.6 |
| *Prescription* |  |  |  |  |  |  |  |  |
| Anti-TB, correct regimen |  |  |  | 0.0 |  |  | 5.6 | 0.1-27.3 |
| Anti-TB, incorrect regimen |  |  |  | 8.3 | 2.8-18.4 |  | 22.2 | 6.4-47.6 |
| Other antibiotics |  |  |  | 58.3 | 44.9-70.9 |  | 38.9 | 17.3-64.3 |
| Anti-TB + other antibiotics |  |  |  | 0.0 |  |  | 0.0 |  |
| Corticosteroids |  |  |  | 28.3 | 17.5-41.4 |  | 11.1 | 1.4-34.7 |
| **Scenario C** |  |  |  |  |  |  |  |  |
| Number of visits (n) |  |  |  | 58 |  |  | 12 |  |
| *Adherence to guideline* |  |  |  |  |  |  |  |  |
| NTP guideline |  |  |  | 81.0 | 68.6-90.1 |  | 66.7 | 34.9-90.1 |
| +CXR |  |  |  | 0.0 |  |  | 0.0 |  |
| *Recommended diagnostic test* |  |  |  |  |  |  |  |  |
| Sputum microscopy |  |  |  | 10.3 | 3.9-21.2 |  | 8.3 | 0.2-38.5 |
| Xpert |  |  |  | 0.0 |  |  | 0.0 |  |
| CXR |  |  |  | 58.6 | 44.9-71.4 |  | 83.3 | 51.6-97.9 |
| *Prescription* |  |  |  |  |  |  |  |  |
| Anti-TB, correct regimen |  |  |  | 12.1 | 4.9-23.3 |  | 25.0 | 5.5-57.2 |
| Anti-TB, incorrect regimen |  |  |  | 5.2 | 1.1-14.4 |  | 0.0 |  |
| Other antibiotics |  |  |  | 20.7 | 11.2-33.4 |  | 16.7 | 2.1-48.4 |
| Anti-TB + other antibiotics |  |  |  | 3.5 | 0.4-11.9 |  | 0.0 |  |
| Corticosteroids |  |  |  | 6.9 | 1.9-16.7 |  | 0.0 |  |
| **Scenario D** |  |  |  |  |  |  |  |  |
| Number of visits (n) | 30 |  |  | 55 |  |  | 11 |  |
| *Adherence to guideline* |  |  |  |  |  |  |  |  |
| NTP guideline | 3.3 | 0.1-17.2 |  | 3.6 | 0.4-12.5 |  | 0.0 |  |
| +CXR | 13.3 | 3.8-30.7 |  | 74.6 | 61.0-85.3 |  | 90.9 | 58.7-99.8 |
| *Recommended diagnostic test* |  |  |  | *0.0* |  |  |  |  |
| Sputum microscopy | 90.0 | 73.5-97.9 |  | 43.6 | 30.3-57.7 |  | 9.1 | 0.2-41.3 |
| Xpert | 3.3 | 0.1-17.2 |  | 3.6 | 0.4-12.5 |  | 0.0 |  |
| CXR | 10.0 | 2.1-26.5 |  | 72.7 | 59.0-83.9 |  | 90.9 | 58.7-99.8 |
| *Prescription* |  |  |  |  |  |  |  |  |
| Anti-TB, correct regimen | 0.0 |  |  | 0.0 |  |  | 9.1 | 0.2-41.3 |
| Anti-TB, incorrect regimen | 0.0 |  |  | 10.9 | 4.1-22.3 |  | 9.1 | 0.2-41.3 |
| Other antibiotics | 6.7 | 0.8-22.1 |  | 60.0 | 45.9-72.9 |  | 36.4 | 10.9-69.2 |
| Anti-TB + other antibiotics | 0.0 |  |  | 1.8 | 0.1-9.7 |  | 0.0 |  |
| Corticosteroids | 6.7 | 0.8-22.1 |  | 20.0 | 10.4-32.9 |  | 0.0 |  |

Abbreviations: CXR: chest x-ray; TB: tuberculosis; NTP: National Tuberculosis Guideline
