# Supplementary material for: Tuberculosis care provided by private practitioners in an urban setting in Indonesia: Findings from a standardized patient study
Source: PLOS Glob Public Health. 2024 Jun 4;4(6):e0003311. doi: 10.1371/journal.pgph.0003311 (PMC11149835; doi:10.1371/journal.pgph.0003311)
Supplement: S2 Table — (DOCX) [file pgph.0003311.s002.docx]

**S2 Table: Clinical enquiries, expressed as percentages and 95% confidence intervals (CIs), performed by different providers under different patient scenarios.**

| Enquiries | Community Health Centers | |  | General Practitioners | |  | Specialists | |
| --- | --- | --- | --- | --- | --- | --- | --- | --- |
|  | % | 95% CI |  | % | 95% CI |  | % | 95% CI |
| **Scenario A** |  |  |  |  |  |  |  |  |
| *History taking* |  |  |  |  |  |  |  |  |
| Presence of cough | 96.7 | 82.8-99.9 |  | 94.2 | 84.1-98.8 |  | 100.0 |  |
| Presence of productive cough | 100.0 |  |  | 86.5 | 74.2-94.4 |  | 93.3 | 68.1-99.8 |
| Amount of sputum | 6.7 | 0.8-22.1 |  | 32.7 | 20.3-47.1 |  | 20.0 | 4.3-48.1 |
| Color of sputum | 46.7 | 28.3-65.7 |  | 46.2 | 32.2-60.5 |  | 60.0 | 32.3-83.7 |
| Whether sputum is bloody | 30.0 | 14.7-49.4 |  | 34.6 | 22.0-49.1 |  | 20.0 | 4.3-48.1 |
| Frequency of cough | 33.3 | 17.3-52.8 |  | 40.4 | 27.0-54.9 |  | 26.7 | 7.8-55.1 |
| Presence of wheezing | 13.3 | 3.8-30.7 |  | 28.8 | 17.1-43.1 |  | 26.7 | 7.8-55.1 |
| Presence of shortness of breath | 40.0 | 22.7-59.4 |  | 42.3 | 28.7-56.8 |  | 73.3 | 44.9-92.2 |
| Presence of chest pain | 30.0 | 14.7-49.4 |  | 32.7 | 20.3-47.1 |  | 40.0 | 16.3-67.7 |
| Presence of fever | 86.7 | 69.3-96.2 |  | 82.7 | 69.7-91.8 |  | 93.3 | 68.1-99.8 |
| Type of fever | 26.7 | 12.3-45.9 |  | 46.2 | 32.2-60.5 |  | 33.3 | 11.8-61.6 |
| Presence of night sweat | 80.0 | 61.4-92.3 |  | 69.2 | 54.9-81.3 |  | 66.7 | 38.4-88.2 |
| Presence of anorexia | 53.3 | 34.3-71.7 |  | 40.4 | 27.0-54.9 |  | 46.7 | 21.3-73.4 |
| Presence of weight loss | 83.3 | 65.3-94.4 |  | 55.8 | 41.3-69.5 |  | 66.7 | 38.4-88.2 |
| Recent history of medication | 70.0 | 50.6-85.3 |  | 67.3 | 52.9-79.7 |  | 53.3 | 26.6-78.7 |
| Names of medicine taken | 46.7 | 28.3-65.7 |  | 57.7 | 43.2-71.3 |  | 53.3 | 26.6-78.7 |
| History of smoking | 36.7 | 19.9-56.1 |  | 40.4 | 27.0-54.9 |  | 53.3 | 26.6-78.7 |
| Alcohol consumption | 0.0 |  |  | 0.0 |  |  | 0.0 |  |
| History of TB | 26.7 | 12.3-45.9 |  | 5.8 | 1.2-15.9 |  | 6.7 | 0.2-31.9 |
| History of TB treatment | 6.7 | 0.8-22.1 |  | 1.9 | 0.0-10.3 |  | 0.0 |  |
| History of DM | 6.7 | 0.8-22.1 |  | 0.0 |  |  | 0.0 |  |
| HIV | 0.0 |  |  | 0.0 |  |  | 0.0 |  |
| History of hypertension | 6.7 | 0.8-22.1 |  | 5.8 | 1.2-15.9 |  | 0.0 |  |
| History of TB in family | 46.7 | 28.3-65.7 |  | 21.2 | 11.1-34.7 |  | 20.0 | 4.3-48.1 |
| *Physical examination* |  |  |  |  |  |  |  |  |
| Pulse rate | 73.3 | 54.1-87.7 |  | 73.1 | 59.0-84.4 |  | 53.3 | 26.6-78.7 |
| Temperature | 23.3 | 9.9-42.3 |  | 28.8 | 17.1-43.1 |  | 13.3 | 1.7-40.5 |
| Blood pressure | 96.7 | 82.8-99.9 |  | 84.6 | 71.9-93.1 |  | 80.0 | 51.9-95.7 |
| Throat examination | 10.0 | 2.1-26.5 |  | 51.9 | 37.6-66 |  | 66.7 | 38.4-88.2 |
| Lymph nodes examination | 3.3 | 0.1-17.2 |  | 25.0 | 14.0-38.9 |  | 33.3 | 11.8-61.6 |
| Chest auscultation | 60.0 | 40.6-77.3 |  | 90.4 | 79.0-96.8 |  | 86.7 | 59.5-98.3 |
| Weight measurement | 73.3 | 54.1-87.7 |  | 25.0 | 14.0-38.9 |  | 26.7 | 7.8-55.1 |
| Height measurement | 20.0 | 7.7-38.6 |  | 0.0 |  |  | 0.0 |  |
| **Scenario B** |  |  |  |  |  |  |  |  |
| *History taking* |  |  |  |  |  |  |  |  |
| Presence of cough |  |  |  | 91.7 | 81.6-97.2 |  | 100.0 |  |
| Presence of productive cough |  |  |  | 80.0 | 67.7-89.2 |  | 94.4 | 72.7-99.9 |
| Amount of sputum |  |  |  | 13.3 | 5.9-24.6 |  | 16.7 | 3.6-41.4 |
| Color of sputum |  |  |  | 45.0 | 32.1-58.4 |  | 77.8 | 52.4-93.6 |
| Whether sputum is bloody |  |  |  | 33.3 | 21.7-46.7 |  | 50.0 | 26.0-74.0 |
| Frequency of cough |  |  |  | 36.7 | 24.6-50.1 |  | 33.3 | 13.3-59.0 |
| Presence of wheezing |  |  |  | 31.7 | 20.3-45.0 |  | 16.7 | 3.6-41.4 |
| Presence of shortness of breath |  |  |  | 53.3 | 40.0-66.3 |  | 44.4 | 21.5-69.2 |
| Presence of chest pain |  |  |  | 43.3 | 30.6-56.8 |  | 22.2 | 6.4-47.6 |
| Presence of fever |  |  |  | 71.7 | 58.6-82.5 |  | 77.8 | 52.4-93.6 |
| Type of fever |  |  |  | 36.7 | 24.6-50.1 |  | 33.3 | 13.3-59.0 |
| Presence of night sweat |  |  |  | 58.3 | 44.9-70.9 |  | 44.4 | 21.5-69.2 |
| Presence of anorexia |  |  |  | 50.0 | 36.8-63.2 |  | 33.3 | 13.3-59.0 |
| Presence of weight loss |  |  |  | 65.0 | 51.6-76.9 |  | 44.4 | 21.5-69.2 |
| Recent history of medication |  |  |  | 65.0 | 51.6-76.9 |  | 33.3 | 13.3-59.0 |
| Names of medicine taken |  |  |  | 46.7 | 33.7-60.0 |  | 27.8 | 9.7-53.5 |
| History of smoking |  |  |  | 33.3 | 21.7-46.7 |  | 50.0 | 26.0-74.0 |
| Alcohol consumption |  |  |  | 0.0 |  |  | 0.0 |  |
| History of TB |  |  |  | 8.3 | 2.8-18.4 |  | 11.1 | 1.4-34.7 |
| History of TB treatment |  |  |  | 3.3 | 0.4-11.5 |  | 5.6 | 0.1-27.3 |
| History of DM |  |  |  | 0.0 |  |  | 5.6 | 0.1-27.3 |
| HIV |  |  |  | 0.0 |  |  | 0.0 |  |
| History of hypertension |  |  |  | 5.0 | 1.0-13.9 |  | 5.6 | 0.1-27.3 |
| History of TB in family |  |  |  | 25.0 | 14.7-37.9 |  | 22.2 | 6.4-47.6 |
| *Physical examination* |  |  |  |  |  |  |  |  |
| Pulse rate |  |  |  | 65.0 | 51.6-76.9 |  | 88.9 | 65.3-98.6 |
| Temperature |  |  |  | 23.3 | 13.4-36.0 |  | 22.2 | 6.4-47.6 |
| Blood pressure |  |  |  | 80.0 | 67.7-89.2 |  | 88.9 | 65.3-98.6 |
| Throat examination |  |  |  | 68.3 | 55.0-79.7 |  | 50.0 | 26.0-74 |
| Lymph nodes examination |  |  |  | 26.7 | 16.1-39.7 |  | 38.9 | 17.3-64.3 |
| Chest auscultation |  |  |  | 98.3 | 91.1-100.0 |  | 94.4 | 72.7-99.9 |
| Weight measurement |  |  |  | 28.3 | 17.5-41.4 |  | 38.9 | 17.3-64.3 |
| Height measurement |  |  |  | 0.0 |  |  | 5.6 | 0.1-27.3 |
| **Scenario C** |  |  |  |  |  |  |  |  |
| *History taking* |  |  |  |  |  |  |  |  |
| Presence of cough |  |  |  | 94.8 | 85.6-98.9 |  | 100.0 |  |
| Presence of productive cough |  |  |  | 74.1 | 61.0-84.7 |  | 83.3 | 51.6-97.9 |
| Amount of sputum |  |  |  | 20.7 | 11.2-33.4 |  | 16.7 | 2.1-48.4 |
| Color of sputum |  |  |  | 46.6 | 33.3-60.1 |  | 58.3 | 27.7-84.8 |
| Whether sputum is bloody |  |  |  | 39.7 | 27.0-53.4 |  | 33.3 | 9.9-65.1 |
| Frequency of cough |  |  |  | 36.2 | 24.0-49.9 |  | 33.3 | 9.9-65.1 |
| Presence of wheezing |  |  |  | 29.3 | 18.1-42.7 |  | 16.7 | 2.1-48.4 |
| Presence of shortness of breath |  |  |  | 43.1 | 30.2-56.8 |  | 16.7 | 2.1-48.4 |
| Presence of chest pain |  |  |  | 34.5 | 22.5-48.1 |  | 8.3 | 0.2-38.5 |
| Presence of fever |  |  |  | 74.1 | 61.0-84.7 |  | 66.7 | 34.9-90.1 |
| Type of fever |  |  |  | 44.8 | 31.7-58.5 |  | 33.3 | 9.9-65.1 |
| Presence of night sweat |  |  |  | 46.6 | 33.3-60.1 |  | 66.7 | 34.9-90.1 |
| Presence of anorexia |  |  |  | 46.6 | 33.3-60.1 |  | 50.0 | 21.1-78.9 |
| Presence of weight loss |  |  |  | 63.8 | 50.1-76.0 |  | 66.7 | 34.9-90.1 |
| Recent history of medication |  |  |  | 62.1 | 48.4-74.5 |  | 25.0 | 5.5-57.2 |
| Names of medicine taken |  |  |  | 44.8 | 31.7-58.5 |  | 16.7 | 2.1-48.4 |
| History of smoking |  |  |  | 19.0 | 9.9-31.4 |  | 50.0 | 21.1-78.9 |
| Alcohol consumption |  |  |  | 0.0 |  |  | 8.3 | 0.2-38.5 |
| History of TB |  |  |  | 10.3 | 3.9-21.2 |  | 33.3 | 9.9-65.1 |
| History of TB treatment |  |  |  | 5.2 | 1.1-14.4 |  | 0.0 |  |
| History of DM |  |  |  | 1.7 | 0.0-9.2 |  | 8.3 | 0.2-38.5 |
| HIV |  |  |  | 0.0 |  |  | 0.0 |  |
| History of hypertension |  |  |  | 3.4 | 0.4-11.9 |  | 8.3 | 0.2-38.5 |
| History of TB in family |  |  |  | 25.9 | 15.3-39.0 |  | 50.0 | 21.1-78.9 |
| *Physical examination* |  |  |  |  |  |  |  |  |
| Pulse rate |  |  |  | 55.2 | 41.5-68.3 |  | 75.0 | 42.8-94.5 |
| Temperature |  |  |  | 22.4 | 12.5-35.3 |  | 33.3 | 9.9-65.1 |
| Blood pressure |  |  |  | 67.2 | 53.7-79 |  | 83.3 | 51.6-97.9 |
| Throat examination |  |  |  | 31.0 | 19.5-44.5 |  | 50.0 | 21.1-78.9 |
| Lymph nodes examination |  |  |  | 12.1 | 5.0-23.3 |  | 58.3 | 27.7-84.8 |
| Chest auscultation |  |  |  | 67.2 | 53.7-79.0 |  | 75.0 | 42.8-94.5 |
| Weight measurement |  |  |  | 31.0 | 19.5-44.5 |  | 33.3 | 9.9-65.1 |
| Height measurement |  |  |  | 1.7 | 0.0-9.2 |  | 8.3 | 0.2-38.5 |
| **Scenario D** |  |  |  |  |  |  |  |  |
| *History taking* |  |  |  |  |  |  |  |  |
| Presence of cough | 96.7 | 82.8-99.9 |  | 98.2 | 90.3-100.0 |  | 100.0 |  |
| Presence of productive cough | 83.3 | 65.3-94.4 |  | 76.4 | 63-86.8 |  | 90.9 | 58.7-99.8 |
| Amount of sputum | 10.0 | 2.1-26.5 |  | 16.4 | 7.8-28.8 |  | 18.2 | 2.3-51.8 |
| Color of sputum | 50.0 | 31.3-68.7 |  | 34.5 | 22.2-48.6 |  | 54.5 | 23.4-83.3 |
| Whether sputum is bloody | 33.3 | 17.3-52.8 |  | 27.3 | 16.1-41.0 |  | 36.4 | 10.9-69.2 |
| Frequency of cough | 36.7 | 19.9-56.1 |  | 25.5 | 14.7-39.0 |  | 36.4 | 10.9-69.2 |
| Presence of wheezing | 16.7 | 5.6-34.7 |  | 27.3 | 16.1-41.0 |  | 36.4 | 10.9-69.2 |
| Presence of shortness of breath | 26.7 | 12.3-45.9 |  | 45.5 | 32.0-59.4 |  | 45.5 | 16.7-76.6 |
| Presence of chest pain | 16.7 | 5.6-34.7 |  | 29.1 | 17.6-42.9 |  | 27.3 | 6.0-61 |
| Presence of fever | 80.0 | 61.4-92.3 |  | 78.2 | 65.0-88.2 |  | 90.9 | 58.7-99.8 |
| Type of fever | 30.0 | 14.7-49.4 |  | 32.7 | 20.7-46.7 |  | 36.4 | 10.9-69.2 |
| Presence of night sweat | 60.0 | 40.6-77.3 |  | 56.4 | 42.3-69.7 |  | 54.5 | 23.4-83.3 |
| Presence of anorexia | 70.0 | 50.6-85.3 |  | 38.2 | 25.4-52.3 |  | 36.4 | 10.9-69.2 |
| Presence of weight loss | 73.3 | 54.1-87.7 |  | 52.7 | 38.8-66.3 |  | 54.5 | 23.4-83.3 |
| Recent history of medication | 46.7 | 28.3-65.7 |  | 72.7 | 59.0-83.9 |  | 45.5 | 16.7-76.6 |
| Names of medicine taken | 40.0 | 22.7-59.4 |  | 40.0 | 27.0-54.1 |  | 18.2 | 2.3-51.8 |
| History of smoking | 20.0 | 7.7-38.6 |  | 23.6 | 13.2-37.0 |  | 36.4 | 10.9-69.2 |
| Alcohol consumption | 0.0 |  |  | 0.0 |  |  | 0.0 |  |
| History of TB | 53.3 | 34.3-71.7 |  | 47.3 | 33.7-61.2 |  | 45.5 | 16.7-76.6 |
| History of TB treatment | 53.3 | 34.3-71.7 |  | 52.7 | 38.8-66.3 |  | 45.5 | 16.7-76.6 |
| History of DM | 0.0 |  |  | 1.8 | 0.0-9.7 |  | 0.0 |  |
| HIV | 0.0 |  |  | 0.0 |  |  | 0.0 |  |
| History of hypertension | 0.0 |  |  | 5.5 | 1.1-15.1 |  | 0.0 |  |
| History of TB in family | 36.7 | 19.9-56.1 |  | 20.0 | 10.4-33 |  | 9.1 | 0.2-41.3 |
| Past sputum test | 46.7 | 28.3-65.7 |  | 63.6 | 49.6-76.2 |  | 63.6 | 30.8-89.1 |
| Past CXR | 20.0 | 7.7-38.6 |  | 70.9 | 57.1-82.4 |  | 36.4 | 10.9-69.2 |
| Past healthcare facility | 76.7 | 57.7-90.1 |  | 78.2 | 65.0-88.2 |  | 81.8 | 48.2-97.7 |
| Past anti-TB treatment | 33.3 | 17.3-52.8 |  | 36.4 | 23.8-50.4 |  | 27.3 | 6.0-61 |
| Past anti-TB treatment duration | 70.0 | 50.6-85.3 |  | 67.3 | 53.3-79.3 |  | 63.6 | 30.8-89.1 |
| Past anti-TB treatment drop out | 70.0 | 50.6-85.3 |  | 61.8 | 47.7-74.6 |  | 45.5 | 16.7-76.6 |
| *Physical examination* |  |  |  |  |  |  |  |  |
| Pulse rate | 56.7 | 37.4-74.5 |  | 54.5 | 40.6-68.0 |  | 81.8 | 48.2-97.7 |
| Temperature | 23.3 | 9.9-42.3 |  | 23.6 | 13.2-37.0 |  | 54.5 | 23.4-83.3 |
| Blood pressure | 90.0 | 73.5-97.9 |  | 87.3 | 75.5-94.7 |  | 100.0 |  |
| Throat examination | 23.3 | 9.9-42.3 |  | 47.3 | 33.7-61.2 |  | 27.3 | 6.0-61.0 |
| Lymph nodes examination | 0.0 |  |  | 18.2 | 9.1-30.9 |  | 36.4 | 10.9-69.2 |
| Chest auscultation | 53.3 | 34.3-71.7 |  | 85.5 | 73.3-93.5 |  | 90.9 | 58.7-99.8 |
| Weight measurement | 46.7 | 28.3-65.7 |  | 25.5 | 14.7-39.0 |  | 45.5 | 16.7-76.6 |
| Height measurement | 23.3 | 9.9-42.3 |  | 0.0 |  |  | 9.1 | 0.2-41.3 |
