# Supplementary material for: Tuberculosis care provided by private practitioners in an urban setting in Indonesia: Findings from a standardized patient study
Source: PLOS Glob Public Health. 2024 Jun 4;4(6):e0003311. doi: 10.1371/journal.pgph.0003311 (PMC11149835; doi:10.1371/journal.pgph.0003311)
Supplement: S3 Table — (DOCX) [file pgph.0003311.s003.docx]

**S3 Table: Diagnosis communicated to standardized patients (SPs) by providers according to scenario.** The total number of working diagnoses may exceed the number of visits as providers may communicate more than one working diagnosis to SPs.

|  | Community Health Centers | |  | General Practitioners | |  | Specialists | |
| --- | --- | --- | --- | --- | --- | --- | --- | --- |
|  | n | % |  | n | % |  | n | % |
| **Scenario A** |  |  |  |  |  |  |  |  |
| Number of visits (n) | 30 |  |  | 52 |  |  | 15 |  |
| TB | 21 | 63.6 |  | 30 | 40.0 |  | 9 | 50.0 |
| Bronchitis | 2 | 6.1 |  | 12 | 16.0 |  | 2 | 11.1 |
| Other LRI | 2 | 6.1 |  | 4 | 5.3 |  | 1 | 5.6 |
| URI | 0 | 0.0 |  | 10 | 13.3 |  | 1 | 5.6 |
| Allergy | 0 | 0.0 |  | 4 | 5.3 |  | 0 | 0.0 |
| Other | 0 | 0.0 |  | 4 | 5.3 |  | 0 | 0.0 |
| No diagnosis | 8 | 24.2 |  | 11 | 14.7 |  | 5 | 27.8 |
| **Scenario B** |  |  |  |  |  |  |  |  |
| Number of visits (n) |  |  |  | 60 |  |  | 18 |  |
| TB |  |  |  | 35 | 44.3 |  | 11 | 47.8 |
| Bronchitis |  |  |  | 7 | 8.9 |  | 2 | 8.7 |
| Other LRI |  |  |  | 3 | 3.8 |  | 1 | 4.3 |
| URI |  |  |  | 10 | 12.7 |  | 2 | 8.7 |
| Allergy |  |  |  | 8 | 10.1 |  | 2 | 8.7 |
| Other |  |  |  | 8 | 10.1 |  | 1 | 4.3 |
| No diagnosis |  |  |  | 8 | 10.1 |  | 4 | 17.4 |
| **Scenario C** |  |  |  |  |  |  |  |  |
| Number of visits (n) |  |  |  | 58 |  |  | 12 |  |
| TB |  |  |  | 57 | 95.0 |  | 11 | 91.7 |
| Bronchitis |  |  |  | 1 | 1.7 |  | 0 | 0.0 |
| Other LRI |  |  |  | 1 | 1.7 |  | 0 | 0.0 |
| URI |  |  |  | 1 | 1.7 |  | 0 | 0.0 |
| Allergy |  |  |  | 0 | 0.0 |  | 0 | 0.0 |
| Other |  |  |  | 0 | 0.0 |  | 0 | 0.0 |
| No diagnosis |  |  |  | 0 | 0.0 |  | 1 | 8.3 |
| **Scenario D** |  |  |  |  |  |  |  |  |
| Number of visits (n) | 30 |  |  | 55 |  |  | 11 |  |
| TB | 27 | 90.0 |  | 40 | 63.5 |  | 7 | 50.0 |
| Bronchitis | 0 | 0.0 |  | 1 | 1.6 |  | 0 | 0.0 |
| Other LRI | 0 | 0.0 |  | 3 | 4.8 |  | 2 | 14.3 |
| URI | 0 | 0.0 |  | 7 | 11.1 |  | 0 | 0.0 |
| Allergy | 0 | 0.0 |  | 0 | 0.0 |  | 1 | 7.1 |
| Other | 0 | 0.0 |  | 1 | 1.6 |  | 0 | 0.0 |
| No diagnosis | 3 | 10.0 |  | 11 | 17.5 |  | 4 | 28.6 |

Abbreviations: LRI: lower respiratory infection; URI: upper respiratory infection; TB: tuberculosis
