# Supplementary material for: Tuberculosis care provided by private practitioners in an urban setting in Indonesia: Findings from a standardized patient study
Source: PLOS Glob Public Health. 2024 Jun 4;4(6):e0003311. doi: 10.1371/journal.pgph.0003311 (PMC11149835; doi:10.1371/journal.pgph.0003311)
Supplement: Attachment — Submitted filename: Response to Reviewers.docx [file pgph.0003311.s004.docx]

We thank the reviewers for their time and thoughtful comments. Please find below our response to the comments.

*Reviewer #1: The topic on TB care in private health care setting is really interesting and the methodology is novel. However, some Major concerns in the study are:
The study was conducted in Bandung city from July 2018 to April 2019 - which is almost before 4 years - so all the guidelines related to TB care has been changed a lot globally. For instance, one of the finding like preference of Chest Xray over sputum examination for TB diagnosis does not remain important - because as per latest guidelines, sputum examination and Chest X-ray are recommended simultaneously for TB diagnosis.

The sample size has been calculated based on Indian study.
The four case scenarios mentioned in the article does not include all situations of TB infection.
All the figures / flow charts are cluttered and not clear.

The manuscript is suitable for national journal.*

Response to reviewer #1:

1. We are aware that in the intervening period between the conduct the study and the present many TB control-relevant contexts had changed, including international and national TB guidelines. Hence, we concur that to some extent the relevance of our findings may have been compromised, especially when referring to the specific example of the use of chest X-ray for screening of pulmonary tuberculosis. On the other hand, we maintain that the general findings and key takeaways still bear importance when considering the roles of private practitioners (PPs) in TB control.

Our study highlighted, and strengthen findings from previous studies, the lack of adherence of PPs to the prevailing guidelines. Other studies have attempted to explore the reasons for this incongruence and pointed to the lack of exposures to the prevailing guidelines and, in cases where PPs were knowledgeable about the guidelines, barriers to following the guidelines (e.g., lack of access to sputum examinations or drugs). Hence, although PPs preference for chest X-ray for TB screening may now be in line with current TB guidelines (of note, this occurred due to changes in the guidelines as opposed to changes in PPs’ behaviour), we are convinced that our findings are applicable even four years after the conduct of the study. This is especially true considering the rapid development of technologies for TB (e.g., diagnostics and drugs, case finding strategies) followed by relatively rapid adoption into policies that potentially widen the knowledge and know-do gaps among PPs.

We have added these messages in our discussion section (lines 411-423).

2. At the time of the study planning, figures from the study by Das et al. were considered more valid for calculating the sample size for our study, especially because relevant local-specific reports were based on surveys or interviews that are prone to social-desirability bias. We were aware that behavioural studies among Indian PPs may not be generalizable to Indonesian PP population. However, considering the low adherence of PPs to local guidelines as found by Das et al, we assume that the numbers could be a conservative estimate for Indonesia due to a more uniform qualification among Indonesian PPs compared to those in India. This assumption was held for the unstratified main outcome measures. Further, although the study was not powered to accurately infer proportions in population subgroups or to identify rarely occurring behaviors, this should not reduce the validity and relevance of the study as it observed PPs behaviors in a natural setting.

We have added these arguments in the discussion section (lines 404-408).

3. Considering the resources needed to execute a standardized patient study, we necessarily restrict our study objective, i.e., to evaluate PP adherence to the national TB guidelines for the management of adult pulmonary TB/presumptive pulmonary TB cases. We would argue that our findings represented “best case scenario” in the management of TB infections/diseases, considering that other forms of TB infections (e.g., latent TB) or disease (e.g., drug-resistant TB) are relatively more complicated compared to pulmonary TB in adults.

We added this in the discussion section (lines 394-399).

4. We have improved the flowchart to the best of our ability.

5. We sincerely disagree with Reviewer #1 with regard to the suitability of our manuscript for a journal with a global audience such as PLOS Global Public Health. While it is true that our study was limited in scope, our study represents the first application of SP method for assessing TB case management practices among PP in Indonesia. Moreover, while Indonesia ranks second globally in terms of TB burden, we are underrepresented in terms of number of scientific publications.^1^ All limitations considered, we are convinced that our study met the highest standards in scientific conduct that merits publication in a well-regarded journal such as PLOS Global Public Health and we therefore strongly believe that it would be in the interest of the global TB audience to have better access to our study report.

^1^Ekenoğlu Merdan Y, Etiz P. A Scopus-Based Bibliometric Analysis of Global Tuberculosis Publications: 1849-2020. Turk Thorac J. 2022 May;23(3):246-256. doi: 10.5152/TurkThoracJ.2022.21284. PMID: 35579232; PMCID: PMC9450016.

*Reviewer #2: This manuscript shows originality and demonstrates good knowledge about the topic. The discussion section is well articulated and relates directly to the results of the study's result and no doubt will add to existing knowledge in the respective field. In addition, the research meets all applicable standards for the ethics of experimentation and research integrity.


However, there are few areas that needed to be updated to make the manuscript ready for submission:
1. The study's goal and objectives are not clearly stated. I personally struggled to understand what the study aimed to achieve - Stating clearly the study's aim and objective will be beneficial to readers and will improve its quality.

2. The method was discussed in details; however, readers are left to figure out the exact methodology used in this study - I suggest you clearly state your methods in addition to providing details to enhance reproducibility. Also, the method and tolls for analysis is not well stated.

3. It does not fully meets the journal's criteria; for instance, there is no section on the conclusion, and the data used for the analysis was not made available - Extracting the conclusion part from the discussion and ensuring that it speaks to the result of the study will be very helpful.

4. Few typos and grammatical errors were observed throughout the sections - There is the need to conduct multiple proof reading before resubmission.*

Response to reviewer #2:

1. We have added the study’s aim in the introduction section (lines 76-81).

2. We have added a brief description about standardized patient method (lines 105-113) and a more detailed information on data analysis and the tools used (lines 185-192).

3. We have added a separate section for conclusion and made the data available.

4. The manuscript has undergone additional proof readings.
